# Population-specific cut-off points of fatty liver index: a study based on the National Health and Nutrition Examination Survey data

**DOI:** 10.1186/s12876-022-02303-z

**Published:** 2022-05-27

**Authors:** Juan Wu, Shen Tian, Hao Li, Zhou Xu, Shu Li, Yu-ling Chen, Xin-yu Liang, Jun Xiao, Jing-yu Song, Rui-ling She, Chen-yu Ma, Jun-han Feng, Zhao-xing Li, Zhi-yu Jiang, Zi-wei Zhang, Kai-nan Wu, Ling-quan Kong

**Affiliations:** 1 Department of Endocrine and Breast Surgery, The First Affiliated Hospital of Chongqing Medical University, Chongqing, 400016 China; 2 Department of Thyroid and Breast Surgery, Affiliated Hospital of North Sichuan Medical College, Nanchong, 637000 Sichuan China

**Keywords:** Fatty liver index, Hepatic steatosis, Cut-off points, Liver ultrasound transient elastography, Controlled attenuation parameter, NHANES

## Abstract

**Background:**

Fatty liver index (FLI) is the most recognized blood biomarker for diagnosis of hepatic steatosis (HS), but lacks the reliable specific cut-off points (COPs). Therefore, we aim to investigate the population-specific COPs of FLI based on the results of liver ultrasound transient elastography (LUTE) and conventional ultrasonography in the National Health and Nutrition Examination Survey (NHANES).

**Methods:**

5948 participants who underwent LUTE from the NHANES 2017–2018 and 14,797 participants who underwent conventional ultrasonography from the Third NHANES (NHANES III) were recruited. FLI was calculated by using body mass index (BMI), waist circumference (WC), triglyceride, and gamma-glutamyl transferase, and its optimal COPs in a specific population (stratified by sex, BMI, and WC) were obtained from receiver operator characteristics (ROC) curve with ultrasonic-diagnosed HS as the reference standard.

**Results:**

Based on LUTE in NHANES 2017–2018, the prevalence of HS and metabolic dysfunction-associated fatty liver disease (MAFLD) were 58.7% and 56.2%, respectively, and the optimal COP of FLI for HS diagnosis in the overall population was 45.60, with an area under ROC curve (AUROC) of 0.833 (0.822–0.844). Based on conventional ultrasonography in NHANES III, the prevalence of HS and MAFLD were 34.4% and 27. 9%, respectively, and the optimal COP of FLI for HS was 59.5, with an AUROC of 0.681 (0.671–0.691). With the increase of BMI and WC, the COPs increased gradually with significant differences between different groups. Compared with conventional ultrasonography, the COPs of FLI based on LUTE were much more precise, with higher diagnostic ability. The population-specific COPs of FLI stratified by gender, WC, and BMI were tabulated.

**Conclusion:**

In the United States, the incidences of HS and MAFLD were high, especially when assessed by LUTE. The FLI based on LUTE is well capable of predicting HS when stratified by gender, WC, and BMI.

**Supplementary Information:**

The online version contains supplementary material available at 10.1186/s12876-022-02303-z.

## Introduction

Along with thorough research, fatty liver disease was recognized to be associated with metabolic dysfunction, and the all-cause mortality of patients with fatty liver disease was much higher than that of the general population [1]. Hence, a new concept, metabolic dysfunction-associated fatty liver disease (MAFLD), was proposed to replace the non-alcoholic fatty liver disease (NAFLD) [2]. The diagnosis of hepatic steatosis (HS) is the first step in both NAFLD and MAFLD. The expert consensus on MAFLD recommended three approaches for the diagnosis of HS: imaging techniques, blood biomarkers/scores, or liver histology.

Fatty liver index (FLI) is a most acknowledged blood biomarker to diagnose HS at present, but no specific COPs were given. FLI is a simple algorithm developed by Bedogni et al. [3] for the prediction of fatty liver in the general population, which is composed of four components: waist circumference (WC), body mass index (BMI), triglycerides (TG), and gamma-glutamyl transferase (GGT). In Bedogni’s studies [3], they concluded that an FLI < 30 can be used to rule out and an FLI ≥ 60 could be used to rule in fatty liver, respectively. However, this COP of FLI for diagnosis of HS was inaccurate due to the absence of stratification of gender and age, although these factors were not considered predictors of fatty liver when FLI was created. Previous studies demonstrated that age and sex were important modifiers of FLI variability [4, 5]. Furthermore, the prevalence of fatty liver was significantly higher in men than that in women with reproductive age, and male gender is a risk factor for fatty liver [6, 7]. Many factors may contribute to gender differences in the incidence of fatty liver, such as waist-to-hip ratio, estrogen, abdominal fat distribution, etc. [8, 9]. Our preliminary study showed significant difference of COPs of FLI for HS diagnosis between Chinese males (37.25: sensitivity = 81.23, AUROC = 0.856) and females (17.00: sensitivity = 85.94, AUROC = 0.909), respectively [10]. In addition to gender stratification, we further provided Asian population-specific COPs of FLI for the diagnosis of HS with stratification of WC and BMI [11]. The COPs of FLI for diagnosing HS varied with different genders, WC and BMI, and so, these factors should be taken into consideration when using FLI to diagnose HS.

The COPs of FLI depend on the results of ultrasonography, a noninvasive first-line modality for the diagnosis of HS. Liver ultrasound transient elastography (LUTE), a new ultrasound technique, could simultaneously measure the ultrasound attenuation related to the presence of HS and records the controlled attenuation parameter (CAP) as the indicator for the fatness in the liver. CAP obtained from LUTE is a numerical value that has been proved to be significantly in line with the percentage of steatosis and the histological degree of steatosis [12–15]. Therefore, the diagnosis of HS by LUTE is much more objective and sensitive than conventional ultrasonography, which contributes to early detection and treatment of HS. In this study, we aimed to obtain the population-specific COPs of FLI based on both conventional ultrasonography and LUTE.

The National Health and Nutrition Examination Survey (NHANES) is a program of studies designed to assess the health and nutritional status of adults and children in the United States. Findings from this survey can be used to assess the health status and disease spectrum of the American population, and used in epidemiological studies and health sciences researches. According to data from the NHANES, in this study, we aimed to obtain the population-specific COPs (stratified by gender, WC and BMI) of FLI based on both conventional ultrasonography and LUTE, so as to better apply FLI for the diagnosis of HS in Americans.

## Materials and methods

### Study design and participants

All study data were collected from the American NHANES (https://www.cdc.gov/nchs/nhanes/index.htm). Participants who underwent LUTE (FibroScan®) (n = 5948) in NHANES 2017–2018 and conventional hepatic ultrasonography (n = 14,979) in NHANES III were enrolled. Invalid or missing data were excluded, and all data were double-checked. Exclusion criteria included: (1) age under 18 years old; (2) without key covariates: BMI, WC, TG and GGT; (3) confidence in HS assessment was “None” or “Doubtful”. After exclusion, the calculation of FLI and determination of COPs were performed on 4633 qualified participants in NHANES 2017–2018 (Additional file 1: Fig. S1) and 9214 participants in NHANES III (Additional file 1: Fig. S2).

### Index calculation

FLI was calculated by BMI (kg/m^2^), WC(cm), TG(mg/dL) and GGT(U/L) of these subjects based on the algorithm [FLI = (e^0.953*loge (triglycerides)+0.139*BMI+0.718*loge(GGT)+0.053*WC−15.745^)/(1 + e^0.953*loge(triglycerides)+0.139*BMI+0.718*loge(GGT)+0.053*WC−15.745^) *100] [3]. Taking the HS diagnosed by ultrasonography as the reference standard, the optimal COPs of FLI were determined in a receiver operating characteristics (ROC) curve analysis by maximizing the Youden index. Homeostatic model assessment for insulin resistance (HOMA-IR) calculated by fasting glucose in mmol/L times insulin in μU/mL divided by 22.5 [16].

### Definitions and subgroups

In NHANES 2017–2018, the presence of HS was determined by LUTE with FibroScan® (EchosensTM North America), and the categorized assessment of HS based on CAP encompassed normal (CAP < 248), mild (248 ≤ CAP < 268), moderate(268 ≤ CAP < 280) and severe (CAP ≥ 280) [17]. HS assessed by hepatic ultrasonography (Toshiba Sonolayer SSA-90A) in NHANES III was also reported as normal, mild, moderate, or severe. Moreover, all mild to severe HS were considered as HS, regardless of the method used. Based on the BMI and WC criteria for Caucasians made by World Health Organization (WHO), BMI was divided into four groups: underweight (< 18.5 kg/m^2^), normal (≥ 18.5 kg/m^2^ and < 25.0 kg/m^2^), overweight (≥ 25.0 kg/m^2^ and < 30.0 kg/m^2^) and obese (≥ 30.0 kg/m^2^), and WC was divided into normal (< 88 cm in female and < 102 cm in male) and abnormal (≥ 88 cm in female and ≥ 102 cm in male) groups. The optimal COPs of FLI for HS diagnosis in different sex were determined for different WC and BMI stratification. MAFLD was defined and diagnosed according to the international expert consensus statement released in 2020 [2]. The history of drug use and various metabolic indicators (HDL-cholesterol, fasting glucose, HbA1c, insulin, high-sensitivity C-reactive protein, etc.) involved in the diagnostic criteria of MAFLD were obtained from the database, and hypertension and diabetes were diagnosed according to widely accepted international standards.

### Statistical analysis

All the continuous variables were tested by normality testing and described by medians (interquartile range), and categorical variables were described by number (proportions). Due to non-normally distributed covariates, the Mann–Whitney U test was performed to compare continuous variables of MAFLD and Non-MAFLD groups, and the Kruskal–Wallis test was used to test the difference among different degrees of HS (mild, moderate and severe). All the analysis was performed at SPSS 26.0. A two-tailed *p* value < 0.05 was considered statistically. The cut-off points of FLI and corresponding sensitivity, specificity, positive likelihood ratio, negative likelihood ratio, Youden index and area under the receiver operating characteristic curve (AUROC) were performed with MedCalc version 19.0.7. The Youden index is sensitivity plus specificity minus 1, and the larger the Youden index, the higher the accuracy of diagnosis. The optimal COPs of FLI for HS diagnosis were determined in a ROC analysis by maximizing the Youden index.

## Results

### Prevalence of HS and baseline characteristics of participants

Finally, a total of 4633 eligible participants in NHANES 2017–2018 and 9214 eligible participants in NHANES III were recruited in this study. In NHANES 2017–2018, the incidence of HS diagnosed by CAP obtained from LUTE in the participants was 58.7% (95% Confidence interval (CI) 57.3–60.1%), with mild, moderate and severe HS accounting for 10.3%, 7.7% and 40.7%, respectively. Overall, the prevalence of HS in males [62.9% (95%CI 60.9–64.9%)] was higher than that in females [54.7% (95%CI 52.7–56.7%), and this difference remained after stratification for BMI and WC. Moreover, with the increase of WC and BMI, the incidence of HS increased significantly. Only 25.86% of participants with normal BMI (≥ 18.5 kg/m^2^ and < 25.0 kg/m^2^) had HS, while 81.68% of overweight participants had HS. Participants with abnormal WC were nearly twice as likely as those with normal WC to have HS. The specific incidence of HS at different WC and BMI stratification were shown in Table 3.

Combined with the participants' medical history and laboratory results, 56.2% (95%CI 54.8–57.6%) had MAFLD. Given the incomplete metabolic indicators of some participants, the prevalence of MAFLD should be higher than 56.2%. Participants with MAFLD had larger BMI and WC, and higher cholesterol, TG, fasting glucose, insulin, HbA1c level and lower high-density lipoprotein cholesterol (Table 1). The level of hepatic enzymes such as aspartate aminotransferase (AST) and alanine aminotransferase (ALT) of participants with MAFLD was significantly higher than that of Non-MAFLD, and the more the severity of HS, the higher the liver stiffness (Table 1). Meanwhile, with the exception of ALT, there was no difference between mild and moderate MAFLD, but there were remarkable differences in all metabolic indicators compared with severe MAFLD (Table 1).

**Table 1:** Baseline characteristic of participants with or without MAFLD in NHANES 2017–2018

	Non-MALFD (n = 2029)	MAFLD (n = 2604)			
		Whole^a^	Mild (n = 429)	Moderate (n = 326)	Severe (n = 1849)
Age (year)	42.0 (33)	55.0 (25)	56.0 (25)	53.5 (30)	55.0 (24)
Gender					
Male	903 (44.5)	1378 (52.9)	189 (44.1)	161 (49.4)	1028 (55.6)
Female	1126 (55.5)	1226 (47.1)	240 (55.9)	165 (50.6)	821 (44.4)
Race-ethnicity					
Mexican American	204 (10.0)	451 (17.3)	53 (12.4)	42 (12.9)	356 (19.3)
Other Hispanic	174 (8.6)	263 (10.1)	56 (13.1)	32 (9.8)	175 (9.5)
Non-Hispanic White	714 (35.2)	892 (34.3)	121 (28.2)	120 (36.8)	651 (35.2)
Non-HISPANIC Black	512 (25.2)	521 (20.0)	108 (25.2)	70 (21.5)	343 (18.6)
Other Race	425 (20.9)	477 (18.3)	91 (21.2)	62 (19.0)	324 (17.5)
BMI (kg/m^2^)	24.6 (6.1)	31.5 (8.3)	29.2 (7.15)	29.4 (6.825)	32.4 (8.7)^cd^
WC (cm)	87.8 (17.3)	106.5 (20.0)	101.0 (16.0)	101.9 (15.9)	109.4 (20.5)^cd^
HC (cm)	97.85 (12.8)	109.0 (17.7)	105.6 (16.5)	106.4 (15.3)	110.4 (18.3)^cd^
ALT (IU/L)	15.0 (9)	20.0 (15)	17.0 (10)	19.0 (12)^b^	22.0 (16)^cd^
AST (IU/L)	19.0 (7)	20.0 (9)	19.0 (8)	19.0 (8)	20.0 (10)^c^
GGT (IU/L)	17.0 (13)	25.0 (21)	20.0 (15)	23.0 (18)	26.0 (24)^cd^
ALB (g/L)	41.0 (4)	41.0 (5)	40.0 (4)	40.0 (4)	41.0 (5)
Cholesterol (mmol/L)	4.63 (1.33)	4.88 (1.42)	4.86 (1.36)	4.84 (1.3)	4.89 (1.42)
Triglyceride (mg/dL)	91.0 (63)	139.0 (99)	118.0 (76)	125.0 (96)	147.0 (102)^cd^
HDLC (mmol/L)	1.45 (0.49)	1.22 (0.42)	1.29 (0.46)	1.24 (0.49)	1.16 (0.39)^cd^
Hs-CRP (mg/L)	1.14 (2.11)	2.62 (4.1)	2.05 (3.52)	2.07 (3.01)	2.87 (4.29)^cd^
Fasting Glucose (mmol/L)	5.00 (0.67)	5.38 (1.11)	5.27 (0.75)	5.27 (0.95)	5.44 (1.27)^cd^
HbA1c (%)	5.4 (0.5)	5.7 (0.8)	5.6 (0.7)	5.6 (0.7)	5.8 (1.0)^cd^
Insulin (μU/mL)	6.96 (5.62)	13.2 (12.6)	10.08 (6.53)	10.17 (9.25)	15.09 (13.7)^cd^
Liver stiffness	4.5 (1.8)	5.4 (2.4)	4.9 (2.0)	5.1 (2.0)	5.6 (2.7)^cd^
CAP (dB/m)	213 (43)	303 (62)	258.0 (10)	273.0 (6)	321.0 (52)

*BMI* body mass index, *WC* waist circumference, *HC* hip circumference, *ALT* alanine aminotransferase, *AST* aspartate aminotransferase, *GGT* aamma glutamyltransferase, *ALB* albumin, *HDLC* high density lipoprotein cholesterol, *CAP* controlled attenuation parameter

^a^All the indexes between Non-MAFLD and MAFLD have statistical differences (*P* < 0.05)

^b^There are statistical difference between mild and moderate MAFLD groups (*P* < 0.05)

^c^There are statistical difference between mild and severe MAFLD groups (*P* < 0.05)

^d^There are statistical difference between moderate and severe MAFLD groups (*P* < 0.05)

In NHANES III, the prevalence of HS and MAFLD was 34.4% (95%CI 33.4–35.3%) and 27.9% (95%CI 27.0–28.8%), respectively. Differences in the prevalence of HS diagnosed by conventional hepatic ultrasonography among various stratifications in NHANES III were similar to NHANES 2017–2018, but much lower than NHANES 2017–2018. And there were statistically significant differences in baseline characteristics among different degrees of HS (Table 2).

**Table 2:** Baseline characteristic of participants with or without MAFLD in NHANES III

	Non-MALFD (n = 6645)	MAFLD (n = 2569)			
		Whole^a^	Mild (n = 853)	Moderate (n = 1006)	Severe (n = 710)
Age (year)	38 (25)	47.0 (27)	43.0 (28)	47.0 (27)^b^	49.0 (26)^c^
Gender					
Male	3021 (45.5)	1269 (49.4)	378 (44.3)	517 (51.4)	374 (52.7)
Female	3624 (54.5)	1300 (50.6)	475 (55.7)	489 (48.6)	336 (47.3)
Race-ethnicity					
Non-Hispanic white	2623 (39.5)	910 (35.4)	287 (33.6)	369 (36.7)	254 (35.8)
Non-Hispanic black	2149 (32.3)	643 (25.0)	247 (29.0)	231 (23.0)	165 (23.2)
Mexican–American	1546 (23.3)	905 (35.2)	278 (32.6)	366 (36.4)	261 (36.8)
Other	327 (4.9)	111 (4.3)	41 (4.8)	40 (4.0)	30 (4.2)
BMI (kg/m^2^)	24.9 (5.9)	29.6 (6.9)	28.7 (7.0)	29.9 (6.9)^b^	30.4 (6.8)^c^
WC (cm)	87.6 (18.0)	101.2 (16.8)	97.4 (16.8)	102.5 (16.4)^b^	103.6 (16.1)^c^
ALT (IU/L)	13.0 (9)	18.0 (14)	15.0 (10)	18.0 (14)^b^	21.0 (18)^cd^
AST (IU/L)	19 (6)	21 (10)	19.0 (8)	21.0 (10)^b^	22.0 (12)^cd^
GGT (IU/L)	20.0 (16)	28.0 (26)	24.0 (21)	29.0 (26)^b^	33.0 (30)^cd^
ALB (g/L)	41 (5)	41 (4)	40.0 (4)	41.0 (4)^b^	41.0 (4)^c^
Cholesterol (mmol/L)	5.02 (1.4)	5.43 (1.45)	5.3 (1.47)	5.48 (1.47)^b^	5.49 (1.35)^c^
Triglyceride (mg/dL)	1.1 (0.78)	1.75 (1.37)	1.55 (1.18)	1.81 (1.39)^b^	1.95 (1.64)^cd^
HDLC (mmol/L)	1.32 (0.49)	1.14 (0.41)	1.19 (0.42)	1.11 (0.39)^b^	1.09 (0.38)^cd^
Fasting Glucose (mmol/L)	5.0 (0.72)	5.38 (1.11)	5.27 (0.94)	5.38 (1.11)^b^	5.55 (1.28)^cd^
HbA1c (%)	5.3 (0.6)	5.5 (0.9)	5.5 (0.7)	5.5 (0.8)^b^	5.6 (1.0)^cd^
Insulin (μU/mL)	7.86 (5.47)	13.35 (10.86)	11.64 (9.31)	13.90 (11.10)^b^	15.18 (12.62)^cd^

*BMI* body mass index, *WC* waist circumference, *HC* hip circumference, *ALT* alanine aminotransferase, *AST* aspartate aminotransferase, *GGT* aamma glutamyltransferase, *ALB* albumin, *HDLC* high density lipoprotein cholesterol

^a^All the indexes between Non-MAFLD and MAFLD have statistical differences (*P* < 0.05)

^b^There are statistical difference between mild and moderate MAFLD groups (*P* < 0.05)

^c^There are statistical difference between mild and severe MAFLD groups (*P* < 0.05)

^d^There are statistical difference between moderate and severe MAFLD groups (*P* < 0.05)

### COPs

In NHANES 2017–2018, the optimal COP of FLI for HS diagnosis in overall population was 45.60, with an AUROC of 0.833 (0.822–0.844), sensitivity of 80.85% (79.3–82.3%) and specificity of 70.50% (68.4–72.5%). For males, if FLI was greater than 48.57, HS could be considered with a sensitivity of 81.80% and specificity of 69.78%. And the value for female to diagnosis HS was 41.93 (sensitivity = 80.34%, specificity = 70.89%). There was a significant difference in the optimal COPs of FLI for HS diagnosis between normal (< 88 cm in females, < 102 cm in males) and abnormal WC groups (35.60 vs. 85.04 in males and 14.29 vs. 75.28 in females). Similarly, the COPs of FLI for HS diagnosis differed greatly by BMI. After stratification by WC and BMI, the COPs of FLI for diagnosis of HS were generally lower in females than those in males. Furthermore, with the increase of BMI and WC, the COPs increased gradually with significant difference between different groups. Detailed data were presented in Table 3. In the underweight group, the diagnostic ability of FLI in diagnosing HS was low, or ROC analysis could not be performed due to insufficient data. However, with the exception of those participants with low body weight, the AUROC of FLI was almost all greater than 0.700, suggesting that FLI had an acceptable diagnostic ability for the diagnosis of HS.

**Table 3:** The cut-off points of FLI stratified by waist circumference and BMI based on the results of liver ultrasound transient elastograpy in NHANES 2017–2018

Stratification	Total	CAP > 248	Prevalence (%)	Cut-off points	AUROC	Sensitivity (true positive fraction) (%)	Specificity (true negative fraction) (%)	Positive likelihood ratio	Negative likelihood ratio	Youden index	*P* value
*Total*	4633	2721	58.73	> 45.60	0.833 (0.822–0.844)	80.85 (79.3–82.3)	70.50 (68.4–72.5)	2.74	0.27	0.5135	< 0.0001
Male	2281	1434	62.87	> 48.57	0.837 (0.821–0.852)	81.80 (79.7–83.8)	69.78 (66.6–72.9)	2.71	0.26	0.5157	< 0.0001
Female	2352	1287	54.72	> 41.93	0.829 (0.813–0.844)	80.34 (78.1–82.5)	70.89 (68.1–73.6)	2.76	0.28	0.5123	< 0.0001
*Stratified by BMI*											
BMI < 18.5	76	7	9.21	> 5.10	0.588 (0.469–0.700)	71.43 (29.0–96.3)	68.12 (55.8–78.8)	2.24	0.42	0.3954	0.5294
18.5 ≥ BMI < 25.0	1191	308	25.86	> 20.77	0.760 (0.734–0.784)	63.31 (57.7–68.7)	78.71 (75.9–81.4)	2.97	0.47	0.4200	< 0.0001
25.0 ≥ BMI < 30.0	1477	863	58.43	> 44.73	0.732 (0.709–0.755)	72.54 (69.4–75.5)	61.56 (57.6–65.4)	1.89	0.45	0.3410	< 0.0001
BMI ≥ 30.0	1889	1543	81.68	> 86.00	0.743 (0.723–0.763)	68.05 (65.7–70.4)	67.92 (62.7–72.8)	2.12	0.47	0.3597	< 0.0001
Male											
BMI < 18.5^a^	31	4	12.90	≤ 1.37	0.611 (0.420–0.780)	50.00 (6.8–93.2)	96.30 (81.0–99.9)	13.5	0.52	0.4630	0.6189
18.5 ≥ BMI < 25.0	554	156	28.16	> 23.26	0.757 (0.719–0.792)	70.51 (62.7–77.5)	73.62 (69.0–77.9)	2.67	0.40	0.4413	< 0.0001
25.0 ≥ BMI < 30.0	814	509	62.53	> 62.09	0.727 (0.695–0.757)	53.05 (48.6–57.5)	80.98 (76.1–85.2)	2.79	0.58	0.3403	< 0.0001
BMI ≥ 30.0	882	765	86.73	> 91.35	0.725 (0.694–0.754)	55.95 (52.3–59.5)	78.63 (70.1–85.7)	2.62	0.56	0.3458	< 0.0001
Female											
BMI < 18.5	45	3	6.67	> 4.68	0.865 (0.730–0.948)	100 (2.92–100.0)	80.95 (65.9–91.4)	5.25	0.00	0.8095	< 0.0001
18.5 ≥ BMI < 25.0	637	152	23.86	> 14.33	0.768 (0.733–0.800)	67.76 (59.7–75.1)	75.05 (71.0–78.8)	2.72	0.43	0.4281	< 0.0001
25.0 ≥ BMI < 30.0	663	354	53.39	> 36.67	0.731 (0.695–0.764)	75.14 (70.3–79.6)	61.49 (55.8–66.9)	1.95	0.40	0.3663	< 0.0001
BMI ≥ 30.0	1007	778	77.26	> 81.83	0.742 (0.714–0.769)	70.95 (67.6–74.1)	66.81 (60.3–72.9)	2.14	0.43	0.3776	< 0.0001
*Stratified by BMI and WC*											
*Male*											
WC < 102	1214	524	43.16	> 35.60	0.780 (0.755–0.803)	74.43 (70.5–78.1)	70.00 (66.4–73.4)	2.48	0.37	0.4443	< 0.0001
BMI < 18.5^a^	31	4	12.90	≤ 1.37	0.608 (0.426–0.771)	50.00 (6.8–93.2)	96.67 (82.8–99.9)	15.0	0.52	0.4667	0.6314
18.5 ≥ BMI < 25.0	552	154	27.90	> 23.26	0.754 (0.716–0.789)	70.13 (62.2–77.2)	73.62 (69.0–77.9)	2.66	0.41	0.4375	< 0.0001
25.0 ≥ BMI < 30.0	572	323	56.47	> 45.60	0.708 (0.669–0.745)	70.90 (65.6–75.8)	59.84 (53.5–66.0)	1.77	0.49	0.3074	< 0.0001
BMI ≥ 30.0	59	43	72.88	> 68.03	0.686 (0.552–0.801)	67.44 (51.5–80.9)	68.75 (41.3–89.0)	2.16	0.47	0.3619	0.0223
WC ≥ 102	1067	910	85.29	> 85.04	0.718 (0.690–0.745)	66.37 (63.2–69.4)	66.24 (58.3–73.6)	1.97	0.51	0.3262	< 0.0001
BMI < 18.5^b^	0										
18.5 ≤ BMI < 25.0^b^	2	0									
25.0 ≤ BMI < 30.0	242	186	76.86	> 63.30	0.679 (0.617–0.738)	69.89 (62.8–76.4)	60.71 (46.8–73.5)	1.78	0.5	0.3061	< 0.0001
BMI ≥ 30.0	823	722	87.73	> 91.35	0.718 (0.686–0.749)	58.86 (55.2–62.5)	75.25 (65.7–83.3)	2.38	0.55	0.3411	< 0.0001
*Female*											
WC < 88	721	157	21.78	> 14.29	0.774 (0.742–0.804)	67.52 (59.6–74.8)	77.48 (73.8–80.9)	3.00	0.42	0.4500	< 0.0001
BMI < 18.5	45	3	6.67	> 4.68	0.865 (0.730–0.948)	100.00 (29.2–100)	80.95 (65.9–91.4)	5.25	0.00	0.8095	< 0.0001
18.5 ≤ BMI < 25.0	526	99	18.82	> 11.58	0.751 (0.711–0.787)	65.66 (55.4–74.9)	74.71 (70.3–78.8)	2.6	0.46	0.4036	< 0.0001
25.0 ≤ BMI < 30.0	146	54	36.99	> 23.48	0.736 (0.657–0.805)	68.52 (54.4–80.5)	70.65 (60.2–79.7)	2.33	0.45	0.3917	< 0.0001
BMI ≥ 30.0^b^	4	1	25.00								
WC ≥ 88	1631	1130	69.28	> 75.28	0.748 (0.726–0.769)	58.23 (55.3–61.1)	78.64 (74.8–82.2)	2.73	0.53	0.3687	< 0.0001
BMI < 18.5^b^	0										
18.5 ≤ BMI < 25.0	111	53	47.75	> 30.65	0.629 (0.532–0.719)	58.49 (44.1–71.9)	68.97 (55.5–80.5)	1.88	0.6	0.2746	0.0171
25.0 ≤ BMI < 30.0	517	300	58.03	> 36.67	0.706 (0.665–0.745)	82.33 (77.5–86.5)	49.77 (42.9–56.6)	1.64	0.35	0.3210	< 0.0001
BMI ≥ 30.0	1003	777	77.47	> 81.83	0.740 (0.711–0.767)	71.04 (67.7–74.2)	66.37 (59.8–72.5)	2.11	0.44	0.3741	< 0.0001

*CAP* controlled attenuation parameter, *AUROC* area under the receiver operating characteristic curves, *BMI* body mass index, *WC* waist circumference

^a^The calculated AUROC < 0.5, which can not meet the requirements of diagnostic test

^b^The optimal cut-off point of FLI could not be obtained due to insufficient data

In NHANES III, the COP of FLI for HS diagnosis in overall participants was 59.54 with an AUROC of 0.681 (0.671–0.691). The COP of FLI for men to diagnose HS was 61.47 (AUROC = 0.706, sensitivity = 55.53%, specificity = 75.15%), and the value for women was 51.65 (AUROC = 0.659, sensitivity = 56.6%, specificity = 71.65%). In NHANES 2017–2018, HS was diagnosed by LUTE, while in NHANES III, it was diagnosed by conventional hepatic ultrasonography. The diagnostic ability of FLI for HS diagnosis in NHANES III was low. Compared with ultrasonography, the COPs of FLI based on LUTE obtained from Fibroscan were much more precise, with higher AUROC, sensitivity and specificity. The population-specific COPs of FLI for HS diagnosis stratified by gender, WC, and BMI were tabulated in Table 4. All COPs were simplistically presented in Tables 5 and 6.

**Table 4:** The cut-off points of FLI stratified by waist circumference and BMI based on the results of conventional ultrasonography in NHANES III

Stratification	Total	Fatty liver	Prevalence (%)	Cut-off points	AUROC	Sensitivity (true positive fraction) (%)	Specificity (true negative fraction) (%)	Positive likelihood ratio	Negative likelihood ratio	Youden index	*P* value
*Total*	9214	3166	34.36	> 59.54	0.681 (0.671–0.691)	55.53 (53.8–57.3)	75.15 (74.0–76.2)	2.23	0.59	0.3068	< 0.0001
Male	4290	1530	35.66	> 61.47	0.706 (0.692–0.720)	58.63 (56.1–61.1)	74.82 (73.2–76.4)	2.33	0.55	0.3345	< 0.0001
Female	4924	1636	33.23	> 51.65	0.659 (0.645–0.672)	56.6 (54.2–59.0)	71.65 (70.1–73.2)	2.00	0.61	0.2826	< 0.0001
*Stratified by BMI*											
BMI < 18.5^a^	190	55	28.95	≤ 3.80	0.582 (0.508–0.653)	76.36 (63.0–86.8)	40.74 (32.4–49.5)	1.29	0.58	0.1710	0.0797
18.5 ≤ BMI < 25.0	3478	831	23.89	> 23.63	0.527 (0.511–0.544)	33.57 (30.4–36.9)	76.39 (74.7–78.0)	1.42	0.87	0.09962	0.0239
25.0 ≤ MI < 30.0	3191	1059	33.19	> 60.84	0.682 (0.666–0.698)	51.46 (48.4–54.5)	76.22 (74.4–78.0)	2.16	0.64	0.2768	< 0.0001
BMI ≥ 30.0	2355	1221	51.85	> 84.10	0.688 (0.669–0.707)	69.04 (66.4–71.6)	60.14 (57.2–63.0)	1.73	0.51	0.2918	< 0.0001
Male											
BMI < 18.5^a^	51	14	27.45	≤ 3.74	0.670 (0.524–0.795)	71.43 (41.9–91.6)	64.86 (47.5–79.8)	2.03	0.44	0.3629	0.0625
18.5 ≤ BMI < 25.0	1632	387	23.71	> 23.75	0.563 (0.538–0.587)	49.10 (44.0–54.2)	64.90 (62.2–67.6)	1.4	0.78	0.1400	0.0004
25.0 ≤ BMI < 30.0	1741	609	34.98	> 60.47	0.683 (0.660–0.704)	61.58 (57.6–65.5)	6.70 (63.9–69.4)	1.85	0.58	0.2827	< 0.0001
BMI ≥ 30.0	866	520	60.05	> 87.90	0.684 (0.652–0.715)	65.77 (61.5–69.8)	63.29 (58.0–68.4)	1.79	0.54	0.2906	< 0.0001
Female											
BMI < 18.5^a^	139	41	29.50	≤ 1.23	0.559 (0.473–0.644)	21.95 (10.6–37.6)	95.92 (89.9–98.9)	5.38	0.81	0.1787	0.2880
18.5 ≤ BMI < 25.0	1846	444	24.05	> 23.65	0.504 (0.481–0.527)	19.82 (16.2–23.8)	89.95 (85.1–88.7)	1.52	0.92	0.06767	0.8171
25.0 ≤ BMI < 30.0	1450	450	31.03	> 45.44	0.679 (0.654–0.703)	60.67 (56.0–65.2)	70.40 (67.5–73.2)	2.05	0.56	0.3107	< 0.0001
BMI ≥ 30.0	1489	701	47.08	> 82.60	0.680 (0.656–0.704)	67.05 (63.4–70.50	61.80 (58.3–65.2)	1.76	0.53	0.2885	< 0.0001
*Stratified by BMI and WC*											
*Male*											
WC < 102	3145	862	27.41	> 47.97	0.624 (0.607–0.641)	46.98 (43.6–50.4)	74.2 (72.4–76.0)	1.82	0.71	0.2118	< 0.0001
BMI < 18.5^a^	51	14	27.45	≤ 3.74	0.670 (0.524–0.795)	71.43 (41.9–91.6)	64.86 (47.5–79.8)	2.03	0.44	0.3629	0.0625
18.5 ≤ BMI < 25.0	1620	382	23.58	> 23.75	0.560 (0.535–0.584)	48.43 (43.3–53.6)	65.27 (62.5–67.9)	1.39	0.79	0.1370	0.0008
25.0 ≤ BMI < 30.0	1353	420	31.04	> 59.10	0.672 (0.647–0.697)	53.57 (48.7–58.4)	72.35 (69.4–75.2)	1.94	0.64	0.2592	< 0.0001
BMI ≥ 30.0	121	46	38.02	> 79.25	0.688 (0.598–0.769)	50.00 (34.9–65.1)	80.00 (69.2–88.4)	2.5	0.63	0.3000	0.0002
WC ≥ 102	1145	668	58.34	> 87.73	0.665 (0.637–0.692)	56.59 (52.7–60.4)	70.23 (65.9–74.3)	1.90	0.62	0.2682	< 0.0001
BMI < 18.5^b^	0										
18.5 ≤ BMI < 25.0	12	5	41.67	> 54.66	0.657 (0.340–0.895)	40.00 (5.3–85.3)	100 (59.0–100.0)	-	0.6	0.4	0.3989
25.0 ≤ BMI < 30.0	388	189	48.71	> 67.07	0.623 (0.573–0.672)	74.60 (67.8–806)	45.23 (38.2–52.4)	1.36	0.56	0.1983	< 0.0001
BMI ≥ 30.0	745	474	63.62	> 88.53	0.653 (0.618–0.687)	67.51 (63.1–71.7)	56.83 (50.7–62.8)	0.56	0.57	0.2434	< 0.0001
*Female*											
WC < 88^a^	2323	552	23.76	≤ 3.46	0.510 (0.489–0.530)	20.11 (16.8–23.7)	86.73 (85.1–88.3)	1.52	0.92	0.06839	0.5030
BMI < 18.5^a^	138	41	29.71	≤ 1.23	0.555 (0.468–0.640)	21.95 (10.6–37.6)	95.88 (89.8–98.9)	5.32	0.81	0.1783	0.3272
18.5 ≤ BMI < 25.0^a^	1663	394	23.69	≤ 3.46	0.510 (0.486–0.535)	20.56 (16.7–24.9)	86.68 (84.7–88.5)	1.54	0.92	0.07241	0.5536
25.0 ≤ BMI < 30.0	489	110	22.49	> 41.89	0.562 (0.516–0.606)	28.18 (20.0–37.6)	89.18 (85.6–92.1)	2.61	0.81	0.1736	0.0697
BMI ≥ 30.0	33	7	21.21	> 27.91	0.599 (0.414–0.765)	100.0 (59.0–100.0)	26.92 (11.6–47.8)	1.37	0.00	0.2692	0.4221
WC ≥ 88	2601	1084	41.68	> 70.28	0.685 (0.667–0.703)	62.18 (59.2–65.1)	65.66 (63.2–68.0)	1.81	0.58	0.2783	< 0.0001
BMI < 18.5^b^	1	0									
18.5 ≤ BMI < 25.0	183	50	27.32	> 35.26	0.552 (0.476–0.625)	44.0 (30.0–58.7)	67.67 (59.0–75.5)	1.36	0.83	0.1167	0.2748
25.0 ≤ BMI < 30.0	961	340	35.38	> 51.64	0.694 (0.664–0.723)	64.12 (58.8–69.2)	66.18 (62.3–69.9)	1.9	0.54	0.3030	< 0.0001
BMI ≥ 30.0	1456	694	47.66	> 82.60	0.676 (0.651–0.700)	67.72 (64.1–71.2)	60.50 (56.9–64.0)	1.71	0.53	0.2822	< 0.0001

*AUROC* area under the receiver operating characteristic curves, *BMI* body mass index, *WC* waist circumference

^a^The calculated AUROC < 0.5, which cannot meet the requirements of diagnostic test

^b^The optimal cut-off point of FLI could not be obtained due to insufficient data

**Table 5:** The cut-off points of FLI at waist circumference and BMI stratifications in NHANES 2017–2018

Gender	Cut-off points	BMI < 18.5	18.5 ≤ BMI < 25.0	25.0 ≤ BMI < 30.0	BMI ≥ 30.0
Whole	45.60	5.1	20.77	44.73	86.00
Male	48.57	–^a^	23.26	62.09	91.35
WC < 102	35.60	–^b^	23.26	45.60	68.03
WC ≥ 102	85.04	–^a^	–^a^	63.30	91.35
Female	41.93	4.68	14.33	36.67	81.83
WC < 88	14.29	4.68	11.58	23.48	–^a^
WC ≥ 88	75.28	–^a^	30.65	36.67	81.83

^a^The optimal cut-off point of FLI could not be obtained due to insufficient data

^b^The calculated AUROC < 0.5, which cannot meet the requirements of diagnostic test

**Table 6:** The cut-off points of FLI at waist circumference and BMI stratifications in NHANES III

Gender	Cut-off points	BMI < 18.5	18.5 ≤ BMI < 25.0	25.0 ≤ BMI < 30.0	BMI ≥ 30.0
Whole	59.54	–^b^	23.63	60.84	84.10
Male	61.47	–^b^	23.75	60.47	87.90
WC < 102	47.97	–^b^	23.75	59.10	79.25
WC ≥ 102	87.73	–^a^	54.46	67.07	88.53
Female	51.65	–^b^	23.65	45.44	82.60
WC < 88	–^b^	–^b^	–^b^	41.89	27.91
WC ≥ 88	70.28	–^a^	35.26	51.64	82.60

^a^The optimal cut-off point of FLI could not be obtained due to insufficient data

^b^The calculated AUROC < 0.5, which cannot meet the requirements of diagnostic test

## Discussion

In this study, we provided, for the first time, detailed population-specific COPs of FLI for the diagnosis of HS among Americans based not only on the common abdominal ultrasonography but also on CAP obtained from LUTE. And all data analyzed were derived from authoritative NHANES. FLI was first proposed by Bedogni et al. for the prediction of fatty liver among Italians (216 subjects with and 280 without suspected liver disease) [3]. Several studies demonstrated that the FLI had excellent discriminative ability to detect ultrasonographic HS, and outperforms other non-invasive markers such as BMI, WC, TG, cholesterol and so on [18–20]. Furthermore, in addition to predicting HS, studies had shown that FLI was also associated with metabolic and cardiovascular disease, and all-cause mortality [21–23]. Therefore, FLI has great clinical application value in health screening and epidemiologic studies, particularly when ultrasound and other imaging examinations are unavailable. However, there were no acknowledged COPs of FLI for the diagnosis of HS. Bedogni et al. suggested that a FLI < 30 could be used to rule out (sensitivity = 87%; specificity = 64%) and a FLI ≥ 60 to rule in hepatic steatosis (sensitivity = 61%; specificity = 86%), respectively. Koehler et al. [20] further validated that the COPs (30 and 60) had great diagnostic efficacy for HS through a Rotterdam study of 2652 participants. A cross-sectional study that included 8626 middle-aged and elderly Chinese (over 40 years old) found the optimal COP of FLI for the diagnosis of HS was also 30 with a maximum Youden Index of 0.51 [24].

However, this COP was inaccurate for that gender and race differences are not taken into account. The COPs of the main components of FLI for HS diagnosis, BMI and WC, were different with the variations of gender and ethnicity. Thus, the COPs of FLI for the diagnosis of HS should be different in different populations and need to be validated when used in a different population. Moreover, NAFLD/MAFLD has been proved to be a heterogeneous disease [25, 26], which makes it more necessary to obtain specific COPs of FLI stratified by heterogeneous factors (age, sex, reproductive status, coexistence of different comorbidities, etc.) for better use in different populations. The gender-based optimal COPs of FLI for HS diagnosis proposed by Motamed et al. were 46.9 (sensitivity = 82.42%, specificity = 76.87%) in men and 53.8 (sensitivity = 82.33%, specificity = 76.55%) in women [19]. In contrast, in the Asian population, the COPs of FLI for HS diagnosis were higher for men than for women, which is in line with the results of Dehnavi et al.’s study [27]. A Taiwan study [18] analyzed the ultrasonography and laboratory results of 29,797 healthy subjects. It concluded that an FLI of < 25 for males & < 10 for females could rule out and an FLI of > 35 for males & > 20 for females could rule in ultrasonographic HS, respectively. Our previous study which recruited 135,436 health check-up populations also showed significant difference between Chinese males and females, with COPs of 37.25 (sensitivity = 81.23, AUROC = 0.856) and 17.00 (sensitivity = 85.94, AUROC = 0.909), respectively. In addition to gender difference, the cut-off values of WC and BMI of males and females are also different. Therefore, our team further obtained the population-specific COPs of FLI, at gender, WC and BMI stratifications, for the diagnosis of HS by using the above physical examination data [11]. The results suggested that, apart from gender, BMI and WC also had a great impact on the COPs of FLI [11]. If the stratifications mentioned above are not considered, only using the fixed COP of FLI to diagnose HS may cause missed diagnosis or misdiagnosis.

Nonetheless, the above researches about the COPs of FLI for HS diagnosis were all based on the common abdominal ultrasonography. It is generally believed that conventional ultrasound is not sensitive to mild fatty infiltration, and only more than 30% of fat infiltration can be accurately detected [28–30]. But, as technology improves, a recent meta-analysis re-evaluated the accuracy of ultrasound in diagnosing hepatic steatosis. Results showed that the overall sensitivity of ultrasonography to detect ≥ 5% histologically defined HS could reach 82% [31]. Ultrasonography is a relatively subjective detecting technology and the results depend on the operator’s experiences. In recent years, a new liver-specific quantitative measurement method, CAP obtained from LUTE, has been developed for HS. CAP uses standardized (controlled) settings, thereby minimizing user influence on the attenuation value, and it can be assessed by an operator who does not have any ultrasound imaging skills. [32] CAP was significantly correlated with the percentage of steatosis [15] and was able to identify steatosis at early stages (> 11%) [32]. A recent study confirmed that the sensitivity of CAP in diagnosing HS was higher than conventional ultrasonography, but without statistical difference. But the difference in specificity of CAP and ultrasonography when using only echogenicity of liver parenchyma of 29% was significant [33]. Hence, the COPs of FLI based on LUTE maybe more accurate than that based on the common ultrasound in the diagnosis of HS. Dehnavi et al. [27] were the first to utilize the fatty liver identified by LUTE to obtain the COPs of FLI, and calculated the COPs of 26.2, 38.3 and 49.7, respectively, in Grades 1, 2, and 3 of HS. However, this study had a small sample size (n = 212) and did not provide criteria for diagnosing HS by CAP nor did further stratification be performed.

To our knowledge, this is the first study to utilize extensive sample size data of the U.S. to provide detailed COPs of FLI, at gender, WC and BMI stratifications, for the diagnosis of HS based on the results of LUTE. According to the Hosmer and Lemeshow guidelines [34] for evaluating predictive abilities, Our study demonstrated that FLI had the excellent diagnostic capability to detect HS with an AUROC of 0.833 (0.822–0.844), sensitivity of 80.85% (79.3–82.3%) and specificity of 70.50% (68.4–72.5%) in the general population. After men and women were divided into two groups, the AUROC could also remain above 0.8, 0.837 (0.821–0.852) in men and 0.829 (0.813–0.844) in women, respectively. Although the diagnostic power of FLI decreased with more detailed stratification, but was still acceptable (AUROC > 0.700). At the same time, the ultrasonography data from NHANES III (1988–1994) was also used to obtain the COPs of FLI, at gender, WC and BMI stratifications, for the diagnosis of HS. The results demonstrated that the prevalence of HS diagnosed by LUTE was much higher than that diagnosed by the common abdominal ultrasonography, and the COPs obtained by LUTE were all smaller than those obtained by the common abdominal ultrasonography in any stratification, which reflected the higher sensitivity of LUTE in the diagnosis of HS from the side.

However, the limitation that cannot be ignored is that the two surveys were performed in different NHANES samples and spanned a long time. In the past 30 years, in addition to the continuous development of ultrasound detection technology, the incidence of HS has been increasing year by year [35–37], which led to the lack of comparability between the two surveys. Another limitation of our study is data processing. NHANES uses a complex, multistage, probability sampling design to select participants representing the civilian, non-institutionalized US population. Therefore, we should have fully considered the sample weights when analyzing NHANES data to account for the complex survey design (including oversampling), survey nonresponse, and post-stratification. In our study, we did not perform weights analysis, and samples with missing data were directly excluded, which made the research results would not be representative of the actual United States. Even so, this study was the first to utilize data from a large sample size cross-sectional survey in the United States to produce detailed FLI COPs with acceptable diagnostic power.

In our study, the COPs of FLI based on CAP obtained from LUTE were much more precise, with higher AUROC, sensitivity and specificity, which suggests that the COPs of FLI for HS diagnosis based on CAP obtained from LUTE was accurate and valuable. Therefore, we recommend to apply the COPs of FLI based on CAP obtained from LUTE in epidemiological investigation and clinical practice when ultrasound is not available. It is worth noting that our study demonstrated the COPs of FLI for the diagnosis of HS varied with different genders, WC and BMI, and so, these factors should be considered when using FLI to diagnose HS. Otherwise, a large number of patients may be missed or misdiagnosed, resulting in a considerable disease burden or waste of medical resources. In this study, relatively accurate and detailed diagnostic COPs of FLI were obtained to apply FLI to clinical practice better. Certainly, further studies are warranted to explore more accurate FLI COPs or other blood biomarkers to diagnose HS.

In conclusion, the present study provided more accurate COPs of FLI based on CAP obtained from LUTE for the diagnosis of HS, and demonstrated that FLI, as a noninvasive, convenient and inexpensive index, has an acceptable ability to predict the occurrence of HS.

## Supplementary Information

**Additional file 1.** Data Screening Flowchart.

## Data Availability

All original data can be obtained from https://www.cdc.gov/nchs/nhanes/index.htm, and the results of the data analysis have been presented in the submitted article.

## Abbreviations

ALT: Alanine aminotransferase
ALB: Albumin
AST: Aspartate aminotransferase
AUROC: Area under the receiver operating characteristic curve
BMI: Body mass index
CAP: Controlled attenuation parameter
COP: Cut-off point
FL: Fatty liver index
GGT: Gamma-glutamyl transferase
HC: Hip circumference
HDLC: High density lipoprotein cholesterol
HS: Hepatic steatosis
LUTE: Liver ultrasound transient elastography
MAFLD: Metabolic dysfunction-associated fatty liver disease
NAFLD: Non-alcoholic fatty liver disease
NHANES: National Health and Nutrition Examination Survey
TG: Triglycerides
WC: Waist circumference

## References

[1] Simon TG, Roelstraete B, Khalili H, Hagström H, Ludvigsson JF (2021). Mortality in biopsy-confirmed nonalcoholic fatty liver disease: results from a nationwide cohort. Gut.

[2] Eslam M, Newsome PN, Sarin SK, Anstee QM, Targher G, Romero-Gomez M, Zelber-Sagi S, Wai-Sun Wong V, Dufour JF, Schattenberg JM (2020). A new definition for metabolic dysfunction-associated fatty liver disease: an international expert consensus statement. J Hepatol.

[3] Bedogni G, Bellentani S, Miglioli L, Masutti F, Passalacqua M, Castiglione A, Tiribelli C (2006). The Fatty Liver Index: a simple and accurate predictor of hepatic steatosis in the general population. BMC Gastroenterol.

[4] Leone A, Battezzati A, Bedogni G, Vignati L, Vanzulli A, De Amicis R, Foppiani A, Bertoli S (2019). Sex- and Age-related differences in the contribution of ultrasound-measured visceral and subcutaneous abdominal fat to fatty liver index in overweight and obese Caucasian adults. Nutrients.

[5] Lonardo ABS, Bedogni G, Bellentani S, Tiribelli C (2021). The Fatty Liver Index (FLI) 15 years later: a reappraisal. Metab Target Organ Damage.

[6] Kim HJ, Lim CW, Lee JH, Park HB, Suh Y, Cho YH, Choi TY, Hwang ES, Cho DK (2016). Gender-based differences in the relationship between fatty liver disease and atherosclerosis. Cardiovasc J Afr.

[7] Vernon G, Baranova A, Younossi ZM (2011). Systematic review: the epidemiology and natural history of non-alcoholic fatty liver disease and non-alcoholic steatohepatitis in adults. Aliment Pharmacol Ther.

[8] de Alwis NM, Day CP (2008). Non-alcoholic fatty liver disease: the mist gradually clears. J Hepatol.

[9] Yang JD, Abdelmalek MF, Pang H, Guy CD, Smith AD, Diehl AM, Suzuki A (2014). Gender and menopause impact severity of fibrosis among patients with nonalcoholic steatohepatitis. Hepatology.

[10] Xu Z, Li H, Tian S, Wu J, Li X, Liu ZL, Li S, Chen YL, Xiao J, Wei JY (2020). Blood biomarkers for the diagnosis of hepatic steatosis in metabolic dysfunction-associated fatty liver disease. J Hepatol.

[11] Wu J, Li H, Xu Z, Ran L, Kong L-Q (2021). Population-specific cut-off points of fatty liver index for the diagnosis of hepatic steatosis. J Hepatol.

[12] Wong GL-H, Wong VW-S (2015). Fat and fiber: how the controlled attenuation parameter complements noninvasive assessment of liver fibrosis. Dig Dis Sci.

[13] Sasso M, Miette V, Sandrin L, Beaugrand M (2012). The controlled attenuation parameter (CAP): a novel tool for the non-invasive evaluation of steatosis using Fibroscan. Clin Res Hepatol Gastroenterol.

[14] de Lédinghen V, Vergniol J, Foucher J, Merrouche W, le Bail B (2012). Non-invasive diagnosis of liver steatosis using controlled attenuation parameter (CAP) and transient elastography. Liver Int Off J Int Assoc Study Liver.

[15] Myers RP, Pollett A, Kirsch R, Pomier-Layrargues G, Beaton M, Levstik M, Duarte-Rojo A, Wong D, Crotty P, Elkashab M (2012). Controlled Attenuation Parameter (CAP): a noninvasive method for the detection of hepatic steatosis based on transient elastography. Liver Int Off J Int Assoc Study Liver.

[16] Wallace TM, Levy JC, Matthews DR (2004). Use and abuse of HOMA modeling. Diabetes Care.

[17] Karlas T, Petroff D, Sasso M, Fan JG, Mi YQ, de Ledinghen V, Kumar M, Lupsor-Platon M, Han KH, Cardoso AC (2017). Individual patient data meta-analysis of controlled attenuation parameter (CAP) technology for assessing steatosis. J Hepatol.

[18] Yang B-L, Wu W-C, Fang K-C, Wang Y-C, Huo T-I, Huang Y-H, Yang H-I, Su C-W, Lin H-C, Lee F-Y (2015). External validation of fatty liver index for identifying ultrasonographic fatty liver in a large-scale cross-sectional study in Taiwan. PLoS ONE.

[19] Motamed N, Sohrabi M, Ajdarkosh H, Hemmasi G, Maadi M, Sayeedian FS, Pirzad R, Abedi K, Aghapour S, Fallahnezhad M (2016). Fatty liver index vs waist circumference for predicting non-alcoholic fatty liver disease. World J Gastroenterol.

[20] Koehler EM, Schouten JNL, Hansen BE, Hofman A, Stricker BH, Janssen HLA (2013). External validation of the fatty liver index for identifying nonalcoholic fatty liver disease in a population-based study. Clin Gastroenterol Hepatol Off Clin Pract J Am Gastroenterol Assoc.

[21] Olubamwo OO, Virtanen JK, Pihlajamaki J, Mantyselka P, Tuomainen T-P (2019). Fatty liver index as a predictor of increased risk of cardiometabolic disease: finding from the Kuopio Ischaemic Heart Disease Risk Factor Study Cohort. BMJ Open.

[22] Gastaldelli A, Kozakova M, Højlund K, Flyvbjerg A, Favuzzi A, Mitrakou A, Balkau B (2009). Fatty liver is associated with insulin resistance, risk of coronary heart disease, and early atherosclerosis in a large European population. Hepatology (Baltimore, MD).

[23] Khang AR, Lee HW, Yi D, Kang YH, Son SM (2019). The fatty liver index, a simple and useful predictor of metabolic syndrome: analysis of the Korea National Health and Nutrition Examination Survey 2010–2011. Diabetes Metab Syndr Obes.

[24] Huang X, Xu M, Chen Y, Peng K, Huang Y, Wang P, Ding L, Lin L, Xu Y, Chen Y (2015). Validation of the fatty liver index for nonalcoholic fatty liver disease in middle-aged and elderly Chinese. Medicine (Baltimore).

[25] Arrese M, Arab JP, Barrera F, Kaufmann B, Valenti L, Feldstein AE (2021). Insights into nonalcoholic fatty-liver disease heterogeneity. Semin Liver Dis.

[26] Eslam M, Sanyal AJ, George J (2020). MAFLD: a consensus-driven proposed nomenclature for metabolic associated fatty liver disease. Gastroenterology.

[27] Dehnavi Z, Razmpour F, Belghaisi Naseri M, Nematy M, Alamdaran SA, Vatanparast HA, Azimi Nezhad M, Abbasi B, Ganji A (2018). Fatty Liver Index (FLI) in predicting non-alcoholic fatty liver disease (NAFLD). Hepat Mon.

[28] Saadeh S, Younossi ZM, Remer EM, Gramlich T, Ong JP, Hurley M, Mullen KD, Cooper JN, Sheridan MJ (2002). The utility of radiological imaging in nonalcoholic fatty liver disease. Gastroenterology.

[29] Sumida Y, Nakajima A, Itoh Y (2014). Limitations of liver biopsy and non-invasive diagnostic tests for the diagnosis of nonalcoholic fatty liver disease/nonalcoholic steatohepatitis. World J Gastroenterol.

[30] Palmentieri B, de Sio I, La Mura V, Masarone M, Vecchione R, Bruno S, Torella R, Persico M (2006). The role of bright liver echo pattern on ultrasound B-mode examination in the diagnosis of liver steatosis. Dig Liver Dis.

[31] Ballestri S, Mantovani A, Byrne CD, Lonardo A, Targher G (2021). Diagnostic accuracy of ultrasonography for the detection of hepatic steatosis: an updated meta-analysis of observational studies. Metab Target Organ Damage.

[32] Sasso M, Beaugrand M, de Ledinghen V, Douvin C, Marcellin P, Poupon R, Sandrin L, Miette V (2010). Controlled attenuation parameter (CAP): a novel VCTE guided ultrasonic attenuation measurement for the evaluation of hepatic steatosis: preliminary study and validation in a cohort of patients with chronic liver disease from various causes. Ultrasound Med Biol.

[33] Runge JH, van Giessen J, Draijer LG, Deurloo EE, Smets A, Benninga MA, Koot BGP, Stoker J (2021). Accuracy of controlled attenuation parameter compared with ultrasound for detecting hepatic steatosis in children with severe obesity. Eur Radiol.

[34] Hosmer DWLS, Sturdivant RX (2013). Applied logistic regression.

[35] Younossi ZM, Koenig AB, Abdelatif D, Fazel Y, Henry L, Wymer M (2016). Global epidemiology of nonalcoholic fatty liver disease-Meta-analytic assessment of prevalence, incidence, and outcomes. Hepatology (Baltimore, MD).

[36] Estes C, Razavi H, Loomba R, Younossi Z, Sanyal AJ (2018). Modeling the epidemic of nonalcoholic fatty liver disease demonstrates an exponential increase in burden of disease. Hepatology (Baltimore, MD).

[37] Cotter TG, Rinella M (2020). Nonalcoholic fatty liver disease 2020: the state of the disease. Gastroenterology.

