# Supplementary material for: Population-specific cut-off points of fatty liver index: a study based on the National Health and Nutrition Examination Survey data
Source: BMC Gastroenterol. 2022 May 27;22:265. doi: 10.1186/s12876-022-02303-z (PMC9145166; doi:10.1186/s12876-022-02303-z)
Supplement: Supplementary file 1 — Additional file 1. Data Screening Flowchart. [file 12876_2022_2303_MOESM1_ESM.docx]

**Additional file 1:** **Data Screening Flowchart**

**Figure S1: Flow chart of inclusion and exclusion criteria in data of NHANES 2017-2018.**

4633 eligible participants were recruited (male: 2281, female:2352)

In NHANES 2017-2018, 6401 participants underwent liver ultrasound transient elastography (LUTE)

453 participants without LUTE results were excluded

226 participants without waist circumference were excluded

226 participants without triglyceride were excluded

1 participants without gamma-glutamyl transferase were excluded

8 participants without body mass index were excluded

715 participants younger than 18 years old were excluded

**Figure S2: Flow chart of inclusion and exclusion criteria in data of NHANES III.**

In NHANES III, 14797 participants underwent conventional ultrasonography

1882 participants who did not have a reliable assessment in hepatic steaosis were excluded

441 participants without waist circumference were excluded

567 participants without triglyceride were excluded

2714 participants without gamma-glutamyl transferase were excluded

9 participants without body mass index were excluded

9214 eligible participants were recruited (male: 4290, female:4924)
